# Response Surface Optimization of Dispersive Solid-Phase Extraction Combined with HPLC for the Rapid Analysis of Multiple Coccidiostats in Feed

**DOI:** 10.3390/molecules27238559

**Published:** 2022-12-05

**Authors:** Haolan Tang, Shudan Liao, Jian Yang, Lilong Zhang, Aijuan Tan, Deyuan Ou, Shiming Lv, Xuqin Song

**Affiliations:** 1 Laboratory of Animal Genetics, Breeding and Reproduction in the Plateau Mountainous Region, Ministry of Education, Guizhou University, Guiyang 550025, China; 2 State Key Laboratory Breeding Base of Green Pesticide & Agricultural Bioengineering, Key Laboratory of Green Pesticide & Agricultural Bioengineering, Ministry of Education, State-Local Joint Laboratory for Comprehensive Utilization of Biomass, Center for R&D of Fine Chemicals, Guizhou University, Guiyang 550025, China; 3 College of Life Science, Guizhou University, Guiyang 550025, China

**Keywords:** coccidiostats, response surface methodology, dispersive solid-phase extraction, high-performance liquid chromatography coupled with diode-array detection, feed

## Abstract

Since antimicrobials were banned as feed additives, coccidiostats with favorable anticoccidial action and growth promotion have been widely used in the breeding industry. The monitoring of coccidiostats in feed is necessary, while the current methods based on mass-spectrometer analysis have limited applicability and matrix effects could interfere with the results. Accordingly, in the present paper, a rapid analytical strategy for the simultaneous determination of six synthetic coccidiostats in feed using high-performance liquid chromatography coupled with diode-array detection was developed. Coccidiostats in chicken feeds were extracted with the trichloroacetic acid–acetonitrile solution. The cleanup was performed by dispersive solid-phase extraction after the optimization of the response surface methodology. The method exhibited good linearity for target coccidiostats within the range of 0.05~20 µg/mL. Recoveries for six compounds in fortified feed samples were from 67.2% to 107.2% with relative standard deviations less than 9.6%. The limit of detection was 0.2~0.3 mg/kg. The successful application of the method in commercial feed verified that it is effective and sensitive for the rapid determination of multiple coccidiostats in chicken feeds.

## 1. Introduction

Coccidiosis is an acute or chronic intestinal disease mainly caused by Eimeria parasitized in the intestinal tract or liver of host animals for a long time. In poultry husbandry, coccidiosis has become a devastating disease because of its fatal mechanical damage to the liver and intestine [1,2]. Once infected with coccidia, poultry suffer from dysentery, bloody diarrhea, weight loss, fever, etc., sometimes accompanied by secondary bacterial infection [3]. The outbreak of coccidiosis is usually massive and rapid, even wiping out an entire flock within a short time [4]. Recent efforts are committed to protecting poultry against coccidiosis, including developing recombinant antigens [5] or DNA vaccines [6]. Some alternatives of coccidiostats are also used, such as probiotics [7], amino acids [8], and plant-active ingredients [9]. Vaccines and alternative substances are safe for poultry, but they cannot meet the expectation of farmers due to the high cost and poor therapeutic effects. Thus, the addition of coccidiostats in animal feed is still the main way to resist the invasion of coccidia at different propagation stages of poultry. 

However, despite the positive impact of coccidiostats on poultry production, the long-term use or the ignorance of withdrawal time may lead to drug residues [10]. Current evidence has proved that coccidiostats applied in animal feed could lead to drug residue in milk or eggs, which presents a potential risk to consumers through the food chain [11]. There is more concern about the emergence of drug resistance caused by prolonged use of coccidiostats [12]. In this case, many governments have established regulations to strictly supervise coccidiostats used as feed additives. Although the European Union has completely banned antimicrobials added to animal feed since 2006, coccidiostats are still authorized as feed additives to protect animals and improve economic benefits [13]. In China, the Ministry of Agriculture and Rural Affairs (MARA) announced the prohibition of antimicrobials in animal feeds in 2020. However, coccidiostats such as clopidol, dinitolmide, sulfaquinoxaline, robenidine, and some ionophore antibiotics are still allowed as chicken feed additives at amounts of 0.05~150 mg/kg, at which the lowest fortified amount of 0.05 mg/kg is hainanmycin because of its toxicity (as shown in Table S1) [14]. The coccidiostats used as feed additives to promote animal growth or control coccidiosis should be under strict supervision because they will become a potential risk when other antimicrobials have been banned. 

The most widely used synthetic coccidiostats include clopidol, dinitolmide, sulfaquinoxaline, robenidine, nicarbazin, and diclazuril (their structures as shown in Figure S1). Clopidol belongs to pyridine compounds and its pKa is 5.50 ± 0.33. Dinitolmide is a nitrobenzamide anticoccidial agent widely used in poultry because of its low toxicity and stability. The pKa of dinitolmide is 3.74 ± 0.50. Sulfaquinoxaline is often used to treat bacterial infections and to prevent coccidiosis, and the pKa of sulfaquinoxaline is 5.65 ± 0.10. Robenidine with a pKa of 5.75 ± 0.70 belongs to a chemical group of guanidines. Nicarbazin is an equimolar complex of 4,4’-dinitrocarbanilide and 4,6-dimethyl-2-pyrimidinol. Diclazuril is a benzene–acetonitrile derivative antiprotozoal, and its pKa is 5.89 ± 0.20. Recent methods for the determination of coccidiostats in feed mainly include liquid chromatography with tandem mass spectrometry (LC-MS/MS) and high-performance liquid chromatography (HPLC). Although LC-MS/MS provides high sensitivity and unambiguous quantification, the complex matrix in animal feed could influence the quantitative results. Pietruk et al. [15] described a sample dilution strategy to minimize the matrix effect. Even so, the responses of diclazuril and clopidol were suppressed significantly under the ESI mode. Moretti et al. [16] concluded that high dilution protocol prevented the ion suppression of coccidiostats, while solid phase extraction (SPE) needed to concentrate the feed sample caused significant matrix effects. As described above, it seems that the high dilution of the feed sample is a simple and effective protocol. When the LC-MS/MS method was used, analytes lost more because of the multiple dilutions. In fact, the HPLC is an alternative for the analysis of coccidiostats because there is less matrix effect. Coccidiostats are commonly used at an excessive dose (mg/kg level) to improve the therapeutic effect, allowing the use of HPLC. More importantly, HPLC is more suitable for the supervision departments in economically backward regions and most pharmaceutical companies. Most literature on the determination of coccidiostats in feed using HPLC coupled with a fluorescence detector (FLD), a diode-array detector (DAD), and an ultraviolet detector (UV) was published in early years [17,18,19]. The complex pretreatment steps described in these early reports cannot meet the current analytical needs. Meanwhile, these methods can only simultaneously analyze a single or two compounds. A study for the HPLC analysis of diclazuril, nicarbazin, and lasalocid in liver based on QuEChERS extraction [20] provided an effective strategy for the determination of coccidiostats. The dispersive solid-phase extraction (DSPE) is a valuable complement to the QuEChERS clean-up procedure. The adsorbent is added into the sample extract to move sugars, pigments, fatty acids or other potential interferents. The DSPE approach was proven to be effective and rapid, because it needs a small amount of adsorbent and solvent [21,22]. As coccidiostats have become the only pharmaceuticals added to feed, it is necessary to control the addition of coccidiostats. To our knowledge, no method has been developed to simultaneously detect coccidiostats in feed authorized by MARA using HPLC. 

This paper aimed to establish a rapid HPLC-DAD method for the analysis of six chemosynthetic coccidiostats authorized by MARA in feed. Since the high concentration of coccidiostats was added in real samples, dilution protocol and DSPE were taken for purification. The response surface methodology was designed to identify the significant variables of DSPE conditions. Finally, the proposed method was applied to the analysis of coccidiostats in feed samples collected from commercial premix and poultry breeding farms. 

## 2. Results and Discussion

### 2.1. Optimization of Chromatographic Conditions

According to the requirements for pharmaceutical feed additives in Bulletin 246 of China, six synthetic coccidiostats with similar UV absorption wavelengths were chosen. The signal response of target drugs at different wavelengths was compared. The results showed that clopidol, diclazuril, and sulfaquinoxaline had the highest signal response at 270 nm. The maximum wavelength of nicarbazin was 340 nm. Although the maximum wavelengths of dinitolmide and robenidine were at 260 nm and 317 nm, respectively, their signal responses at 270 nm reduced slightly. Therefore, the double wavelength of 270 nm and 340 nm were selected for HPLC analysis.

A conventional reversed-phase C_18_ column combined with the mobile phase of methanol (MeOH), acetonitrile (ACN), ammonium acetate (NH_4_OAc), and formic acid (FA) can achieve ideal retention and efficient separation for coccidiostats [15,20,23]. Three kinds of C_18_ columns including Agilent Extend-C_18_ (250 mm × 4.6 mm i.d., 5 μm), Agilent Zorbax SB-C_18_ (150 × 4.6 mm i.d., 5 μm), and Welch Ultimate XB-C_18_ (250 × 4.6 mm i.d., 5 μm) were investigated. The results (Figure S2) showed that the Agilent Extend-C_18_ provided symmetry and sharp peak and no peak tailing, resulting from its unique double-liganded bonded phase and end-capped performance.

**Figure 1:**
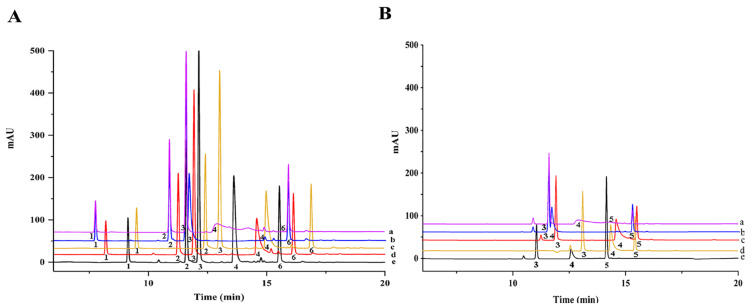
Effects of different compositions of mobile phase on the HPLC chromatogram of coccidiostats at 270 nm (**A**) and 340 nm (**B**): 1, clopidol; 2, dinitolmide; 3, sulfaquinoxaline; 4, robenidine; 5, nicarbazin; 6, diclazuril. The composition of mobile phase: a (pink colour), acetonitrile-water; b (blue colour), methanol-water; c (red colour), methanol-5 mmol/L ammonium acetate aqueous solution; d (yellow colour) , methanol-10 mmol/L ammonium acetate aqueous solution; e (black colour), methanol-5 mmol/L ammonium acetate aqueous solution (containing 0.1% formic acid).

MeOH and ACN were commonly used as an organic phase in HPLC analysis. In this study, MeOH provided better separation and peak shape than ACN. The aqueous phase including different concentrations of NH_4_OAc (0, 5 and 10 mmol/L) was optimized to decrease secondary retention. As presented in Figure 1, broad shape, tailing peak and low response were observed under the mobile phase of water and MeOH. The participation of NH_4_OAc in the aqueous phase improved the responses and peak shapes for most coccidiostats. However, the tailing peak of robenidine occurred when 10 mmol/L NH_4_OAc was used. In addition, the addition of FA in the aqueous phase improved the sensitivity and peak shape of robenidine. The 0.1% FA in NH_4_OAc aqueous solution provided a favorable response and sharp peak for target analytes.

### 2.2. Sample Preparation

**Figure 2:**
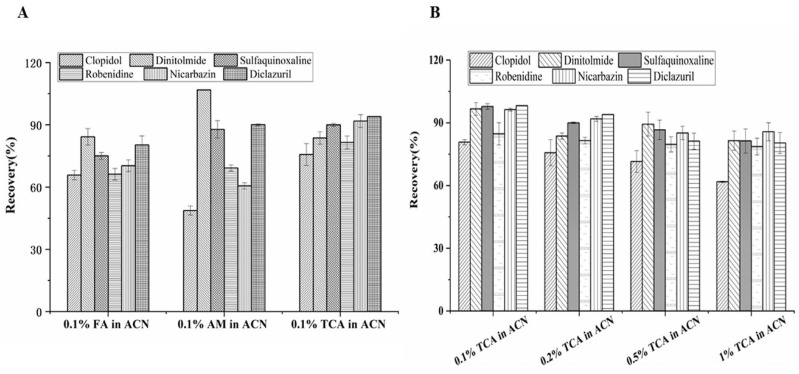
Effects of (**A**) different kinds of extraction solvents and (**B**) various concentrations of TCA in ACN on recoveries of target compounds, with the abbreviation defined as the follows: TCA, trichloroacetic acid; ACN, acetonitrile; FA, formic acid; AM, ammonium (n = 3).

MeOH and ACN were commonly used to extract coccidiostats [15,20]. Many impurities were co-extracted from the chicken feed when MeOH was used. Although ACN could reduce the co-extracted impurities, the recoveries of nicarbazin and robenidine were lower than 60%, perhaps because of their strong affinity with feed. The acidified and alkalized ACN were optimized to obtain favorable recoveries of six coccidiostats. As shown in Figure 2A, alkalized ACN (0.1% AM in ACN) could not effectively extract clopidol and nicarbazin, with their recoveries lower than 60%. As a protein denaturant, trichloroacetic acid (TCA) improves the release of coccidiostats from feed samples by changing protein conformation and precipitating proteins. However, the more TCA was used, the lower recovery of clopidol showed (Figure 2B). When the ratio of TCA reached 1%, the recovery of clopidol was only 61.8%, suggesting that the clopidol could degrade dramatically under low pH. It needs further study to assess how the pH affects the stability of clopidol. Therefore, ACN containing 1% FA was selected as the extract solution.

**Figure 3:**
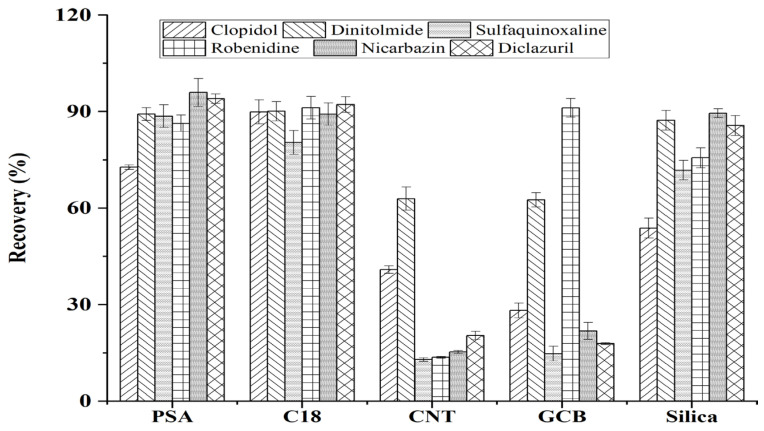
The influence of different commonly used adsorbents including primary secondary amine (PSA), C_18_, graphitized carbon black (GCB), carbon nanotube (CNT), and silica on the recoveries of target compounds (n = 3).

The complex ingredients of animal feed such as fat, protein, vitamin, minerals, and plant fiber could affect the extraction efficiency. Considering that coccidiostats are commonly spiked to feed at a high level in the entire growing period of chicken, a DSPE protocol combined with dilution was taken. The DSPE protocol has the advantages of fast purification, strong adsorption, and wide availability, becoming an alternative way in the analysis of trace compounds from animal-derived food [24] and feed [25]. This study compared five adsorbents including primary secondary amine (PSA), carbon nanotube (CNT), C_18_, graphitized carbon black (GCB), and silica. The results are given in Figure 3. Due to the large adsorption surface area of CNT, CNT adsorbent provided low recoveries for most analytes, especially for robenidine, nicarbazin, and diclazuril (lower than 10%). Since GCB has a high affinity to nitrogen-containing heterocyclic compounds, the recoveries of clopidol, sulfaquinoxaline, nicarbazin, and diclazuril were below 50%. Although the C_18_ adsorbent obtained a satisfactory recovery for each analyte, the purification was poorer than PSA because the C_18_ can only remove non-polar impurities. The recoveries of analytes obtained by PSA were lower than C_18_, while the PSA exhibited better purification. Due to the favorable ion exchange ability of PSA, it has excellent purification performance to polar or nonpolar impurities in feed such as fatty acid, sugar, and chelate metal ions. Then, because of the high recovery of C_18_ and the powerful purification of PSA, a response surface methodology was performed to optimize the amount and proportion.

**Figure 4:**
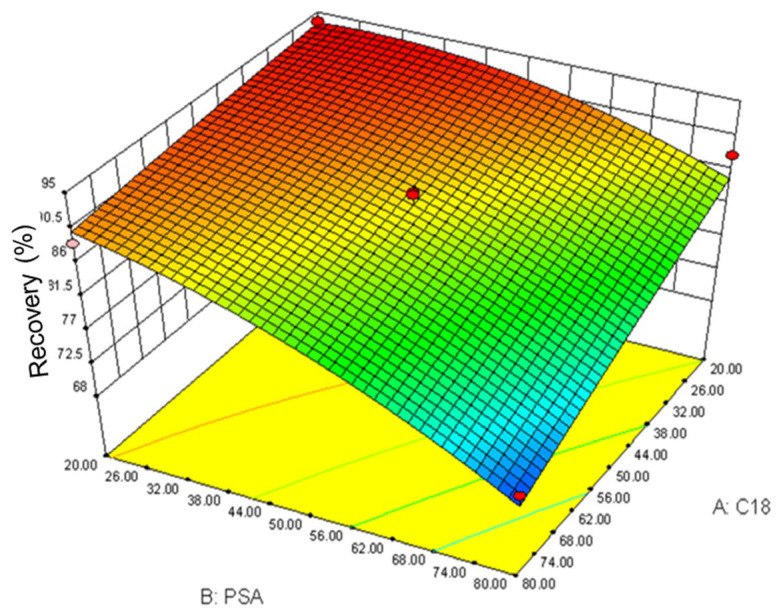
The three-dimensional overall desirability response surface plot for the PSA and C_18_ factors under the CCD test.

Response surface methodology has the advantages of fewer experiments, high accuracy, and good model predictability, which is widely applied in the removal of pollutants [26] and the extraction of active ingredients [27]. To obtain the optimal conditions of DSPE, a center combinatorial design (CCD) test for two factors (the mass of C_18_ as factor A and PSA as factor B) was carried out to investigate the interaction between the variables. The CCD test contained 13 random experiments. The design and results of 13 random experiments are given in Table S2. The three-dimensional overall desirability (D) response surface plot for the factors is exhibited in Figure 4. The optimum conditions were: 50 mg of C_18_ and 50 mg of PSA, and the predicted recovery was 87.5%. The recovery test was repeated three times under the optimal conditions and the result was consistent with the predicted value, indicating that the conditions obtained by the response surface methodology were reliable.

To achieve efficient reconstitution, different proportions of MeOH in 5 mM NH_4_OAc aqueous solution (5%, 20%, 50% and 80%) were evaluated. As exhibited in Figure S3, 5% MeOH in 5 mM NH_4_OAc aqueous solution is unable to completely re-dissolve nicarbazin and diclazuril, with their recoveries of less than 30%. The MeOH improved the recoveries of nicarbazin and diclazuril. The more MeOH in the reconstitution solution, the higher recoveries were obtained. When 80% MeOH in 5 mM NH_4_OAc aqueous solution was used as a reconstitution solution, the highest recovery for each analyte was achieved.

### 2.3. Method Validation

The linearity of the proposed method was evaluated by calibration curves plotted with the concentrations of 0.05, 0.1, 1, 5, 10, and 20 µg/mL. The calibration curves revealed good linearity in the ranges of 0.05~20 µg/mL for clopidol, diclazuril, and nicarbazin, and 0.1~20 µg/mL for other analytes. The linear regression coefficient (*r*^2^) was higher than 0.99 (regression equations omitted). 

**Figure 5:**
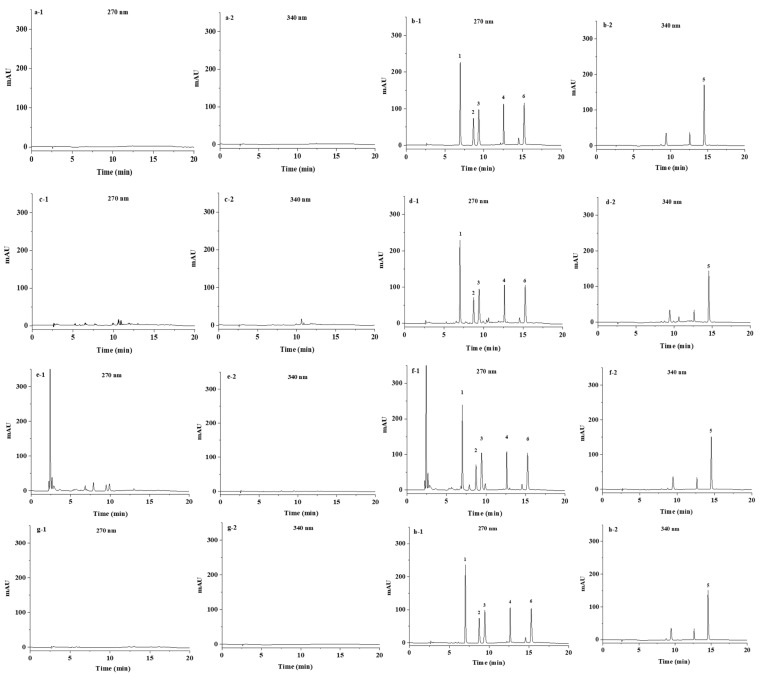
Typical HPLC chromatograms at 270 nm and 340 nm of (**a**) reagent blank, (**b**) reference solution (2 μg/mL), (**c**) blank matrix of chicken premix, (**d**) spiked chicken premix at the concentration of 20 mg/kg, (**e**) blank matrix of chicken complete feed, (**f**) spiked chicken complete feed at the concentration of 20 mg/kg, (**g**) blank matrix of chicken feed additive, and (**h**) spiked chicken feed additive at the concentration of 20 mg/kg, with peak identification as follows: 1, clopidol; 2, dinitolmide; 3, sulfaquinoxaline; 4, robenidine; 5, nicarbazin; 6, diclazuril.

The recovery was estimated by spiking 1, 10, and 20 mg/kg of coccidiostats in three kinds of chicken feeds. The intra-day relative standard deviation (RSD) is calculated by analyzing quality control samples in six replicates within one day, and inter-day RSD is evaluated by repeating each spiked level within three consecutive days. As summarized in Table 1, the mean recoveries of six analytes at three spiked concentrations were from 67.2% to 107.2%, which could meet the requirement of routine analysis. The intra-day and inter-day RSDs were observed at less than 9.5% and 9.7%, respectively. The HPLC chromatograms of blank feed and spiked feed samples are shown in Figure 5. Thanks to the clean-up strategy of DSPE and dilution, no impurity interference was observed around the retention time of target analytes.

The limit of detection (LOD) and limit of quantification (LOQ) of target analytes in three feed samples were in ranges of 0.2~0.3 mg/kg and 0.5~1.0 mg/kg, which were far below the allowable concentrations in chicken feed approved by the MARA. The results of method validation indicated that the developed method was reliable and sensitive for the content assay of coccidiostats in chicken feed.

### 2.4. Stability

Due to the diverse physicochemical properties of coccidiostats, the stability of these analytes in pure solvent and sample solution should be considered to ensure the accuracy of the analysis. As given in Figure S4, 6 coccidiostats are stable in pure solvent and sample solution within a week at 4 °C. However, except for diclazuril, most coccidiostats tended to degrade sharply at 25 °C after one day. There was no apparent degradation for diclazuril during evaluation time, demonstrating the favorable stability of diclazuril. The result agrees with the finding that diclazuril has a long half-life of more than 119 days under room temperature [28]. Thus, reconstitutes were suggested to store at 4 °C in the dark, and the analysis should be performed as soon as possible.

### 2.5. Applications to Real Samples

To verify the applicability of the proposed method, 50 commercial chicken feeds including complete feed, premix, and feed additive were analyzed. The premix feeds detected were free from target analytes. In six complete feeds, robenidine, clopidol, and diclazuril were detected, ranging from 0.44 mg/kg (robenidine) to 7.06 mg/kg (diclazuril), indicating the frequent use of these compounds in complete feeds for therapy or prevention purpose. In addition, the contents of eight premix feeds were analyzed lower than that of the label indication. Considering the extensive and long-term use of coccidiostats in chicken feeds, strict monitoring approaches should be adopted.

## 3. Materials and Methods

### 3.1. Reagents and Materials

HPLC grade solvents of methanol, acetonitrile, formic acid, and trichloroacetic acid were bought from Kermel Chemical Reagents Development Center (Tianjin, China). Ammonium acetate and ammonium were purchased from Thermo Fisher Scientific (Fairlawn, NJ, USA). Ultrapure water was taken by a Millipore MilliQ system (Molsheim, France). SPE adsorbents including primary secondary amine were purchased from CNW Technologies GmbH (Düsseldorf, Germany), carbon nanotube from Nanjing XFNANO Materials Tech Co., Ltd. (Nanjing, China), graphitized carbon black, C_18_, and silica from Shanghai Welch Materials, Inc. (Shanghai, China).

### 3.2. Standards and Stock Solutions

Reference standards (purity higher than 95%) of clopidol, dinitolmide, sulfaquinoxaline, robenidine, nicarbazin, and diclazuril were purchased from LGC Labor GmbH (Augsburg, Germany). The stock solution of dinitolmide (1 mg/mL) was prepared by weighing 10 mg and dissolved by 10 mL of MeOH. Clopidol, sulfaquinoxaline, robenidine, nicarbazin, and diclazuril were completely dissolved with 0.5 mL of DMSO and then diluted with ACN to 10 mL to prepare the stock solution (1 mg/mL). Stock solutions were stored at −20 °C in the dark for no more than 6 months. Mixed standard solutions (100 μg/mL) were prepared with 1 mL of each stock solution and diluted with ACN to 10 mL. Working standard solution was prepared by diluting mixed standard solutions daily with the mixture of MeOH and 5 mmol/L NH_4_OAc aqueous solutions (50:50, *v/v*) containing 0.5% FA.

### 3.3. Sample Preparation

Chicken feed samples including complete feed, premix, and feed additive were purchased from local feed markets (Guiyang, China). After grinding and sifting by a 60-mesh sieve, feed samples were stored at room temperature.

The main components of chicken feed used in methodological development included corn, wheat, bean, fish meal, bone meal, vitamins, and minerals. Chicken feed (1 g ± 0.01 g) was weighed into a 15 mL polypropylene centrifuge tube and spiked with the appropriate working standard solution. Samples were left for 30 min at room temperature to ensure the dispersion of analytes into feed before proceeding. Samples were ultrasonically extracted with 5 mL of 0.1% TCA in ACN solution for 10 min and mechanically shaken for 20 min. After centrifugation, the residue performed the extract again and the supernatant was combined. A total of 1 mL of the supernatant was transferred to a 2 mL polypropylene centrifuge tube. Then, 50 mg of PSA and 50 mg of C_18_ were used to purify impurities. The solution was vortexed for 1 min and centrifuged at 10,000 rpm for 2 min. The supernatant was evaporated to dryness under nitrogen at 45 °C and residues were reconstituted with 1 mL of MeOH-5 mmol mL^−1^ NH_4_OAc containing 0.5% FA aqueous solution (80:20, *v/v*) for the HPLC analysis.

### 3.4. Response Surface Methodology

To check the optimal performance of the single variable method, preliminary tests were carried out to evaluate the effects of different adsorbents (PSA, CNT, GCB, C_18_, and silica) on purification. Considering the important role of different factors in the DSPE procedure, significant variables including adsorbents, amounts, and recovery were investigated by a center combinatorial design (CCD) test using Design-Expert 8.0.6 software. The relationship between the recovery and independent variable was clarified, and finally, the optimal conditions of purification were obtained.

### 3.5. HPLC Analysis

Instrumental analysis was performed on the Agilent Technologies 1260 series HPLC (Agilent Technologies, Santa Clara, CA, USA) system equipped with a DAD. The separation was carried out using an Agilent Extend-C_18_ column (250 mm × 4.6 mm i.d., 5 μm). The mobile phase was composed of MeOH (mobile phase A) and 5 mmol/mL NH_4_OAc aqueous solution containing 0.1% formic acid (mobile phase B). The gradient elution program was as follows: 0 min, 5% A; 1 min, 5% A; 3 min, 50% A; 6 min, 80% A; 14 min, 50% A; 17 min, 5% A; and 20 min, 5% A, with a constant flow rate of 1 mL/min. The UV absorption wavelength was set to 270 nm and 340 nm. The injection volume was 20 μL. It is noted that nicarbazin contains equal amounts of 4,4′-dinitrodiphenylurea and 4,6-dimethyl-2-hydroxypyrimidine. In this study, the quantification of nicarbazin has calculated the sum of two components, which conforms to the national standard of the determination of nicarbazin in feeds (GB/T 19423-2020).

## 4. Conclusions

This paper described a rapid and reliable method for the simultaneous determination of six authorized chemosynthetically coccidiostats in chicken feeds using HPLC-DAD combined with a DSPE procedure. The response surface methodology was designed to achieve purification conditions. The method provided high linearity, sensitivity, and accuracy, suggesting the feasibility of this method for the routine assay of coccidiostats contents in chicken feed. From the determination results from real samples, coccidiostats were usually added to chicken feed while some premixes did not meet the quality standards. More effective measures should be taken to improve the quality standards of production, and more importantly, the determination of coccidiostats in chicken feeds should be reinforced.

## Figures and Tables

**Table 1:** Recovery and precision of six coccidiostats in feed samples (n = 6) *^a^*.

Compound	Feed	Intra-Day Recovery (RSD), %			Inter-Day Recovery (RSD), %		
		1 mg/kg	10 mg/kg	20 mg/kg	1 mg/kg	10 mg/kg	20 mg/kg
Clopidol	A	91.9(3.9)	90.3(4.0)	98.1(1.8)	91.8(3.8)	90.6(5.4)	93.2(5.7)
	B	95.0(5.2)	103.5(5.6)	97.7(2.9)	77.7(8.9)	89.2(9.3)	94.8(5.9)
	C	67.1(3.7)	74.0(9.4)	77.9(4.9)	77.6(9.5)	81.2(9.6)	83.6(6.5)
Dinitolmide	A	85.5(2.6)	92.6(2.9)	96.7(6.3)	91.8(5.4)	91.7(4.6)	90.9(5.2)
	B	86.1(3.7)	87.8(5.2)	94.8(2.7)	86.0(6.6)	93.9(7.8)	91.8(5.4)
	C	83.7(7.7)	90.8(7.5)	91.7(4.6)	92.2(9.3)	90.1(6.6)	90.9(5.2)
Sulfaquinoxaline	A	87.5(3.3)	82.1(2.1)	87.8(3.7)	87.5(3.3)	82.4(5.3)	89.4(5.3)
	B	92.4(5.4)	107.2(6.1)	97.9(6.1)	85.0(8.9)	88.6(9.6)	92.7(5.1)
	C	67.9(5.4)	67.2(7.6)	70.6(6.2)	68.4(2.4)	70.2(5.3)	72.6(6.2)
Robenidine	A	87.2(3.8)	76.9(6.5)	92.4(2.3)	90.7(7.4)	76.4(8.2)	91.4(6.1)
	B	82.3(7.3)	81.0(3.9)	86.7(2.9)	73.4(6.8)	76.5(9.6)	74.4(6.2)
	C	82.5(9.5)	79.0(9.4)	93.9(6.3)	83.2(8.0)	81.8(9.5)	86.8(9.6)
Nicarbazin	A	83.0(3.9)	73.9(2.6)	84.1(4.5)	86.7(6.3)	75.1(7.5)	86.6(5.5)
	B	68.7(8.2)	72.9(7.0)	84.2(3.4)	72.0(6.8)	83.5(8.9)	82.2(4.9)
	C	80.6(6.6)	76.6(6.4)	86.1(4.6)	82.2(7.9)	77.4(8.2)	83.1(7.1)
Diclazuril	A	95.6(3.6)	86.1(6.4)	84.7(6.2)	91.2(6.8)	88.8(7.6)	83.6(6.2)
	B	103.2(8.4)	84.4(8.2)	85.5(6.8)	86.3(9.0)	85.8(9.7)	87.3(6.5)
	C	97.6(8.4)	78.7(7.6)	77.8(3.6)	88.0(8.8)	82.0(8.9)	80.4(8.2)

*^a^* A, complete feed; B, premix; C, feed additive; RSD, relative standard deviation.

## Data Availability

Not applicable.

## Supplementary Materials

The following supporting information can be downloaded at: https://www.mdpi.com/article/10.3390/molecules27238559/s1, Figure S1: Effect of different C_18_ columns on the separation of coccidiostats: 1, clopidol; 2, dinitolmide; 3, sulfaquinoxaline; 4, robenidine; 5, nicarbazin; 6, diclazuril; Figure S2: Effect of different proportion of MeOH in 5 mM NH_4_OAc aqueous solution on the recoveries of target analytes (n = 3); Figure S3: The stability of six coccidiostats in chicken complete feed matrix (20 mg/kg) stored at 4 °C and room temperature (25 °C); Table S1: The contents of coccidiostats in chicken premix and complete feed allowed by the Ministry of Agriculture and Rural Affairs of China.
