# Supplementary material for: Response Surface Optimization of Dispersive Solid-Phase Extraction Combined with HPLC for the Rapid Analysis of Multiple Coccidiostats in Feed
Source: Molecules. 2022 Dec 5;27(23):8559. doi: 10.3390/molecules27238559 (PMC9738599; doi:10.3390/molecules27238559)
Supplement: Supplementary file 1 [file molecules-27-08559-s001.zip › molecules-2054332-supplementary.pdf]

Supplementary Materials

Response Surface Optimization of Dispersive Solid-Phase Extraction Combined with HPLC for the Rapid Analysis of Multiple Coccidiostats in Feed

Haolan Tang [1,†], Shudan Liao [1,†], Jian Yang [1], Lilong Zhang [2], Aijuan Tan[3], Deyuan Ou [1], Shiming Lv [1,*] and Xuqin Song [1,*]

[1] Laboratory of Animal Genetics, Breeding and Reproduction in the Plateau Mountainous Region, Ministry of Education, Guizhou University, Guiyang 550025, China
[2] State Key Laboratory Breeding Base of Green Pesticide & Agricultural Bioengineering, Key Laboratory of Green Pesticide & Agricultural Bioengineering, Ministry of Education, State-Local Joint Laboratory for Comprehensive Utilization of Biomass, Center for R&D of Fine Chemicals, Guizhou University, Guiyang 550025, China
[3] College of Life Science, Guizhou University, Guiyang 550025, Guizhou Province, China
* Correspondence: lvlvsm@163.com (S.L.); song1991yi@163.com (X.S.)
† The authors contributed equally to this work.

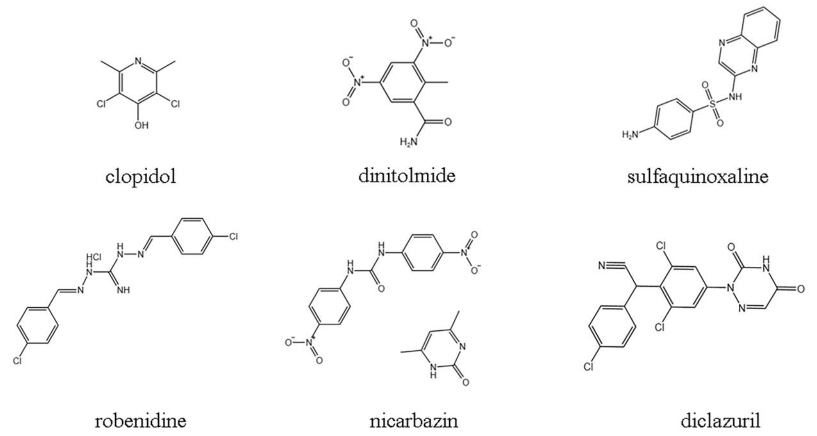

Figure S1. Chemical structures of six coccidiostats.

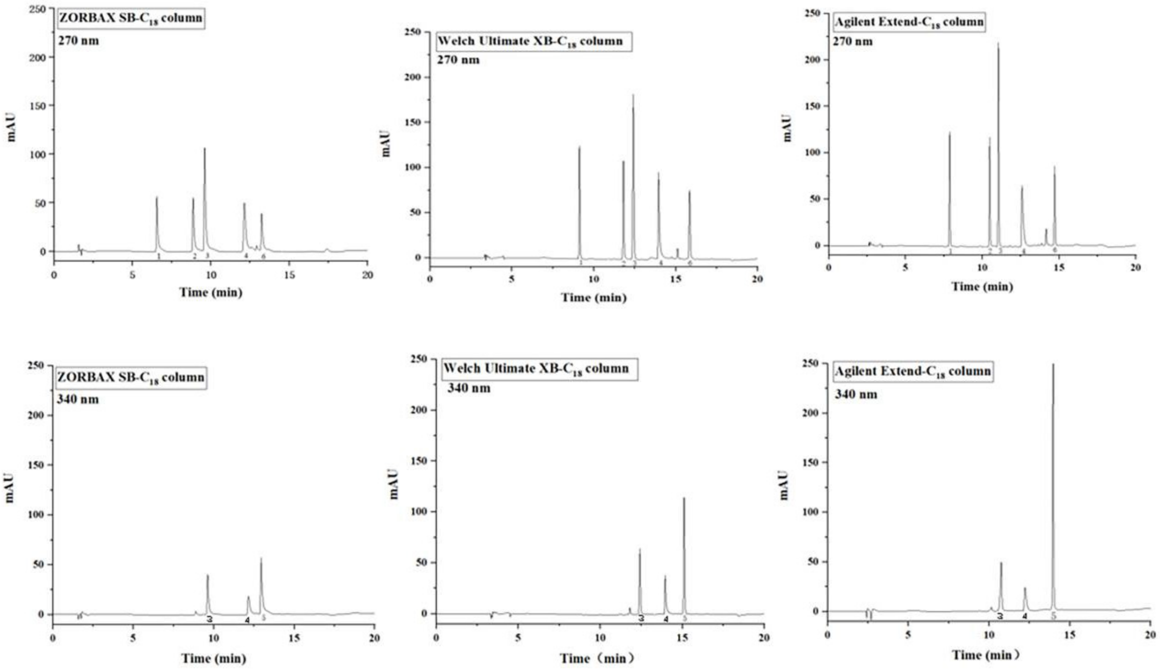

Figure S2. Effect of different C18 columns on the separation of coccidiostats: 1, clopidol; 2, dinitolmide; 3, sulfaquinoxaline; 4, robenidine; 5, nicarbazin; 6, diclazuril.

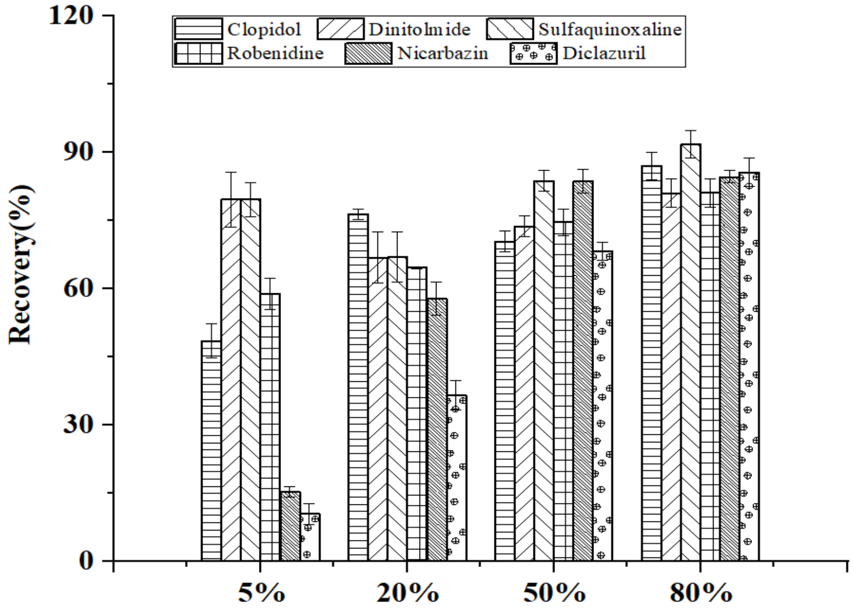

Figure S3. Effect of different proportion of MeOH in 5 mM NH_4OAc aqueous solution on the recoveries of target analytes (n=3).

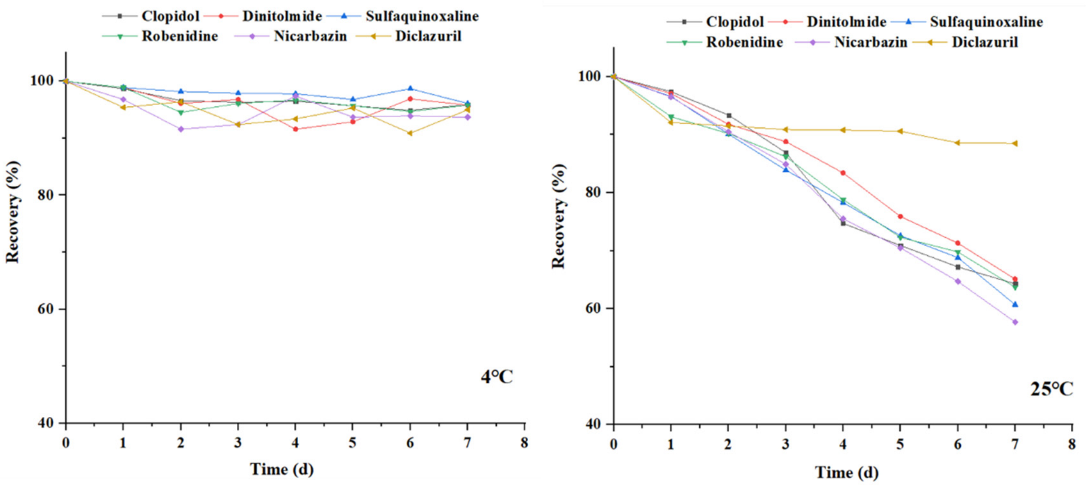

Figure S4. The stability of six coccidiostats in chicken complete feed matrix (20 mg/kg) stored at 4 °C and room temperature (25 °C).

Table S1. The contents of coccidiostats in chicken premix and complete feed allowed by the Ministry of Agriculture and Rural Affairs of China

Compound	Premix (g/kg)	Complete feed (mg/kg)
Dinitolmide	250	150
Maduramicin	10	5
Robenidine	100	30~60
Amprolium	250	150
Sulfaquinoxaline	120	60
Hainanmycin	10	0.05~0.075
Clopidol	250	150
Diclazuril	5	1
Salinomycin	100	6
nicarbazin	250	25~31
Monensin	100	9~11

Table S2. The design and results of 13 random experiments in CCD test for C_{18} and PSA adsorbents (n=3)

Run number	PSA (mg)	C_{18} (mg)	Recovery±SD (%)
1	50	50	86.7±0.079
2	50	7.6	93.6±0.010
3	50	50	88.7±0.083
4	50	50	86.6±0.086
5	20	80	88.0±0.017
6	80	80	84.4±0.013
7	80	20	88.7±0.012
8	50	50	89.8±0.085
9	50	92.4	86.9±0.041
10	7.6	50	92.1±0.078
11	20	20	93.7±0.038
12	92.4	50	80.6±0.014
13	50	50	86.2±0.008
